# Summary of the Impact of the Inclusion of Mobile Phone Numbers into the NSW Population Health Survey in 2012

**DOI:** 10.3934/publichealth.2015.2.210

**Published:** 2015-06-02

**Authors:** Margo Barr, Raymond Ferguson, Jason van Ritten, Phil Hughes, David Steel

**Affiliations:** 1. Centre for Epidemiology and Evidence, NSW Ministry of Health, 73 Miller Street, North Sydney, NSW 2060, Australia; 2. National Institute for Applied Statistics Research, University of Wollongong, NSW 2522, Australia

**Keywords:** CATI survey, mobile phone, overlapping dual-frame

## Abstract

**Background:**

Although it was estimated that 20% of the population in Australia were mobile-only phone users in 2010, the inclusion of mobile numbers into computer-assisted telephone interviews (CATI) behavioural risk factor surveys did not occur until 2012.

**Methods:**

Three papers have been published describing the methods, weighting strategy and the impact in detail of including mobile numbers into the NSW Population Health Survey (NSWPHS). This paper identifies the important components of those papers and summarises them for a broader audience.

**Results:**

In the 2012 NSWPHS, 15,214 (15,149 with weights) interviews were completed (64% landline frame; 36% mobile frame). Response, cooperation and contact rates were 37%, 65% and 69% respectively. The inclusion of mobile phone numbers resulted in a sample that was closer to the NSW population profile and impacted on the time series of estimates for alcohol drinking, recommended fruit consumption, current smoking, and overweight or obesity.

**Conclusions:**

The papers found that including mobile phone numbers into NSWPHS did not impact negatively on response rates or data collection, but it did cost more and affect the time series for some behavioural risk factors, in that it corrected the estimates that had been produced from a sample frame that was progressively getting less representative of the population.

## 1. Introduction

In Australia most statewide health behaviour surveillance was undertaken using telephone surveys and landline phone frames [1]–[6] until 2012 when mobile phone numbers were included into the NSW Population Health Survey (NSWPHS) using an overlapping dual-frame design [7]. The NSWPHS had been operating since 1997 to collect information on health behaviours, health status and health service to monitor the health of the population, and for policy development and program evaluation. The survey has approval from the NSW Population and Health Services Research Ethics Committee [1].

In 2012 the landline phone sample procedures were kept the same as in previous years using random digit dialing (RDD) derived from prefixes associated with NSW postcodes. The sample was stratified by health administration area and one person was selected from each household [1]. The mobile phone sample procedures however needed to be developed.

The existing weighting strategy for the NSWPHS, as with most sample surveys, involved determining of the probability of selection, calculation of the weights and then benchmarking to population information, in this case by age, sex and stratum [1]. Modification however needed to occur to account for the mobile phone sample.

Three papers have been written to describe in detail the impact of the inclusion of mobile phone numbers into the 2012 NSWPHS; specifically the impact on the sampling, data collection, call outcomes, costs, the representativeness of the resultant sample, weighting and the time series [7]–[9].

## 2. Materials and Method

### 2.1. Collection

In the 2012 NSWPHS, 15,214 (15,149 with weights) interviews were completed (64% landline frame; 36% mobile frame). Response, cooperation and contact rates were 37%, 65% and 69% respectively as described in Barr et al 2012 [7].

The final mobile frame procedure consisted of RDD numbers being generated from the provider prefixes and valid numbers being identified. Calls were made until a NSW resident was identified (only one third of the numbers). The responder or mobile phone owner was selected. In order to ensure that children of people who did not have a landline were also included, when respondent's had one or more children one child was also selected at random [7].

### 2.2. Weighting

The new weighting strategy included an additional step, involving averaging the estimates for the dual-phone users obtained from the two frames. The ratio of phone numbers in the sample to phone numbers in the population (for each frame) also needed to be calculated which was not needed when only a single frame had been used. Information on the number of phone numbers for each frame was available for Australia, but not for NSW and so it was necessary to impute them. For the mobile frame children also needed to be associated to their parents and weighted accordingly. Further details about the required changes to the weighting strategy are provided in Barr et al [8].

### 2.3. Time series

Health indicators *drank five or more drinks of alcohol in a day, drank more than two alcoholic drinks in a day, met the recommended fruit intake, met the recommended vegetable intake, were current smokers, did adequate physical activity, had positive self-rated health status, had current asthma, were ever diagnosed with diabetes, and were overweight or obese were selected*. For further details on the questions that were used for each indicator see Barr et al 2014 [9]. Prevalence estimates and 95 percent confidence intervals were calculated for each indicator using the SURVEYFREQ procedure in SAS, which uses the Taylor expansion method to calculate sampling errors of complex sample designs [10].

Estimates for health related variables for the 2012 NSWPHS, as well as using just the landline frame sample, re-benchmarked to the NSW population, were then compared to the 2011 NSWPHS estimates. Statistically significant differences were identified by comparing the differences between the two estimates, divided by the standard error of the differences, calculated as √ [SE(E_2011PHS_)^2^ + SE(_E2012PHS_)^2^] ), with the standard normal distribution [11]. The full time series was examined for current smoking and overweight and obesity because there were significant differences between 2011 and 2012.

## 3. Results

### 3.1. How did it impact on collection?

In Barr et al 2012 [7] data from the first quarter of 2012 NSWPHS were used to test the weighting strategy. This consisted of data on 3,395 respondents with 2,171 (63.9%) being from the landline frame of which 382 (17.6%) were landlines-only and 1,224 (36.1%) being from the mobile frame of which 316 (25.8%) were mobile-only.

**Table 1.** Summary of call outcome, productivity and cost by frame, quarter 1 2012 NSWPHS.

Parameter		Mobile frame	Landline frame
Details	Complete Interview	2171	1224
	Survey length	15.8	17.2
Outcome	Response	31.5%	35.1%
	Co-operation	72.8%	71.4%
	Refusal	11.7%	14.0%
	Contact	62.9%	68.0%
Productivity	Calls to get a contact	2.1	1.9
	Calls to get an eligible contact	10.5	7.0
	Calls to get an interview	14.4	9.8
Cost	Call cost per completed interview	$38.90	$7.45
	Interviewer costs per completed interview	$35.53	$23.68
	Total costs per completed interview	$74.42	$31.13

When the call outcomes, using American Association for Public Opinion Research (AAPOR) definitions, [12] for the first quarter of 2012 NSWPHS were compared between frames, mobile frame, compared to the landline frame as shown in Table 1, response rates were the same, refusal rates were lower, because of the higher level of unknowns, contact rates were lower, because of the lack of geography on the mobile frame and co-operation rates were slightly higher.

When productivity and costings for the first quarter of 2012 NSWPHS were compared between frames, completed interviews from the mobile frame, compared to the landline frame, were slightly shorter, cost 2.3 times more for each completed interview and required more telephone numbers to obtain a contact, eligible contact and an interview [7].

When the sample for the first quarter of the 2012 NSWPHS dual frame (with adjustment for the dual phone-users overlap) was compared to the NSW demographic profile [7], as shown in Table 2, it was only significantly different for age group whereas the landline frame was different for age group, sex, country of birth, marital status and income.

**Table 2.** Summary of sample comparisons to the latest population profile for NSW.

Demographic group	p-values for the difference to 2011 Census			
	Landline frame	Mobile frame	Landline plus mobile only	Both frames combined #
Age group	< 0.001 *	0.03 *	< 0.001 *	0.01 *
Sex	0.04 *	0.85	0.07	0.20
Aboriginality	0.86	0.94	0.96	0.96
Country of birth	0.02 *	0.42	0.007	0.30
Marital status	< 0.001 *	0.76	0.01 *	0.08
Income	0.02 *	0.04 *	0.02 *	0.05

NOTE: *significantly (*p* < 0.05) different to the 2011 Census. #with adjustment for the dual phone-users overlap.

### 3.2. How did it impact on weighting?

The final sampling and weighting strategy was as follows: within a stratum the landline sample was selected using equal probability of selection of landline telephone numbers and then random selection of one person from the selected household. In the mobile phone sample an equal probability sample of mobile telephone numbers in Australia was selected and screened for adult residents in NSW. If the respondent had one or more children one child was selected at random. Sample weights thus reflected the differing sampling probabilities. The sample weights of the dual phone-users were then adjusted so that the composite factor used to combine the estimates for this component obtained from the landline sample and the mobile phone sample, λ, was set at 0.5. Benchmarking to the reference population was then performed by adjusting the weights for differences between weighted estimates of the age and sex structure obtained from the combined landline and mobile phone sample and ABS mid-year population estimates for each stratum. Further details about the weighting strategy are provided in Barr et al 2014 [8].

In Barr et al 2014 [8] data from the first quarter of 2012 NSWPHS were used to assess the weighting strategy. This consisted of data on 3378 respondents who had all core weighting variables (age, sex, stratum, number of landline phones, number of mobile phones they personally have, and eligible persons in the household) and 2933 adults and 445 children, had sufficient data to be included. On average person weights were 3.3 times higher for the mobile-only respondents, 1.3 times higher for the landline-only respondents and 1.7 times higher for dual-phone users in the mobile frame compared to the dual-phone users in the landline frame. The overall weight effect for the first quarter of 2012 was 1.93 and the coefficient of variation of the weights was 0.96. The weight effects for 2012 were similar to, and in many cases less than, the effects found in the corresponding quarter of the 2011 NSWPHS when only a landline based sample was used.

### 3.3. How did it impact on the time series?

In Barr et al 2014 [9] it was estimated from the dual frame 2012 NSWPHS that 11.1% of the population *drank five or more drinks of alcohol in a day*, 27.6% *drank more than two alcoholic drinks in a day*, 53.4% *met the recommended fruit intake*, 10.0% *met the recommended vegetable intake*, 17.1% *were current smokers*, 56.2% *did adequate physical activity*, 82.4% *had positive self-rated health status*, 10.1% *had current asthma*, 8.4% *were ever diagnosed with diabetes*, and 49.7% *were overweight or obese*. When these health indicator estimates from the 2012 NSWPHS were compared to the 2011 NSWPHS, when samples were only taken from the landline frame, significantly higher estimates were found in 2012 for: *recommended fruit intake, recommended vegetable intake, current smoking, positive self-reported health status*, and significantly lower estimates for *overweight or obese* as shown in Table 3.

**Table 3.** Summary of health indicators estimate comparisons by year, NSWPHS.

Health Indicator	2011 NSWPHS%	2012 Dual frame%	2012 Landline frame%	p-value diff	
				2012 Dual frame minus 2011	2012 Landline frame minus 2011
Five or more drinks of alcohol in a day	11.3	11.1	9.4	0.432	0.083
More than two alcoholic drinks in a day	29.6	27.6	27.1	0.092	0.042 #
Recommended fruit intake	50.4	52.4	55.9	0.016 *	< 0.001 *
Recommended vegetable intake	8.4	10.0	12.3	0.026 *	< 0.001 *
Current smokers	14.7	17.1	14.4	0.011 *	0.373
Adequate physical activity	54.6	56.2	56.8	0.224	0.069
Positive self-rated health status	80.3	82.4	80.6	0.010 *	0.381
Current asthma	11.3	10.1	12.6	0.079	0.122
Ever diagnosed with diabetes	8.1	8.4	8.6	0.573	0.215
Overweight or obese	52.2	49.7	53.9	0.047 #	0.138

NOTE: *significantly (*p* < 0.05) higher than comparison group; #significantly (*p* < 0.05) lower than comparison group.

Also as shown in Table 3 when these health indicators estimates were calculated using just the landline frame sample for 2012, re-benchmarked to the NSW population were compared to the 2011 NSWPHS significantly higher estimates were again found for *recommended fruit intake* and *recommended vegetable intake*, and significantly lower estimates for *more than two alcoholic drinks in a day*. However *current smoking*, *positive self-reported health status*, and *overweight or obese* were no longer statistically significantly different, and the difference had changed in direction for *current smoking* and *overweight or obese*.

**Figure 1.**
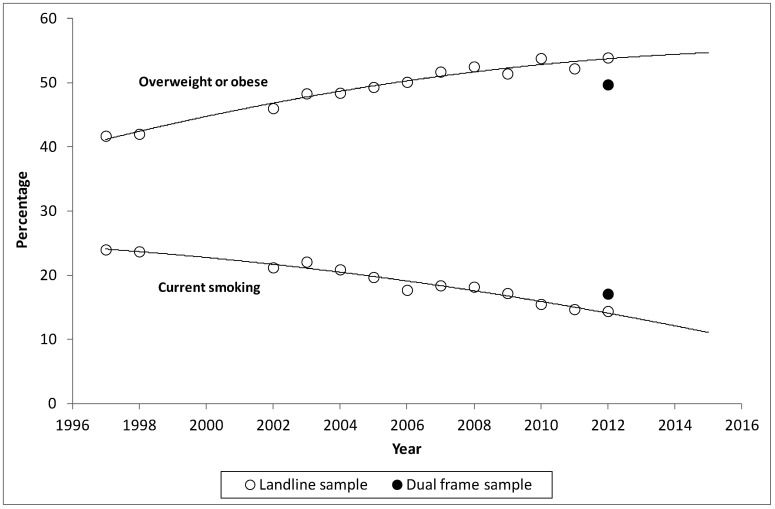
Landline sample time series estimates for current smoking and overweight or obese from the NSWPHS compared to the estimates from the dual-frame for 2012 NSWPHS.

The two indicators for which the time series was most likely to be affected, as identified by Barr et al 2014 [9] were current smoking and overweight or obese. Looking at the full time series, as shown in Figure 1, if the NSWPHS had continued to be undertaken only using a landline frame, *overweight or obese* would have been shown to continue to increase and *current smoking* would have been shown to continue to decrease. However, with the introduction of the overlapping dual-frame design in 2012, *overweight or obese* increased until 2011 and then decreased in 2012, and *current smoking* decreased until 2011, and then increased in 2012.

## 4. Discussion

The inclusion of the mobile phone number was logistically very challenging with the biggest challenge being the lack of geography on the mobile frame which resulted in more time and resources being spent on calling ineligible numbers (persons who reside outside NSW). The inclusion of mobile phone numbers in the NSWPHS however is still important to do because of the additional interviews that were conducted with young people, Aboriginal and Torres Strait Islanders and people who were born overseas resulting in a more representative sample. This however may not be the case for smaller states where the cost of excluding ineligible (out of state) persons may be prohibitive.

The development of the weighting strategy, weighted for the person selection probabilities by frame, composite weights applied to dual-phone users, and benchmarked to the NSW population, was more complex than it had been for the previous landline frame. It was however encouraging that the weight effects were similar to those found in the previous year, when only a landline based sample was used.

Type of phone-use was associated with many of the health indicators, in particular mobile-only phone users were significantly different for: *drink five or more drinks of alcohol in a day, current smoking, recommended vegetable intake,* and *overweight or obese*, even after adjusting for the weighting variables. These results were consistent with other studies [13]–[15].

The inclusion of the mobile telephone numbers through an overlapping dual-frame design did impact on the time series for *current smoking* and *overweight or obese* in that the changes were a consequence of the sampling frame change rather than between years. However the sampling design change corrected the estimates that were being calculated from a sample frame, which was getting progressively less representative of the population.

## 5. Conclusion

In conclusion, the impact of including of mobile phone numbers into the NSW Population Health Survey in 2012 was substantial but manageable. However, not including mobile phone numbers would have meant that the resultant health estimates would progressively get “further from the truth” because of the increasing coverage error.
